# Two-Dimensional Shear Wave Elastography versus Transient Elastography: A Non-Invasive Comparison for the Assessment of Liver Fibrosis in Patients with Chronic Hepatitis C

**DOI:** 10.3390/diagnostics10050313

**Published:** 2020-05-16

**Authors:** Rosanna Villani, Francesco Cavallone, Antonino Davide Romano, Francesco Bellanti, Gaetano Serviddio

**Affiliations:** C.U.R.E. (University Centre for Liver Disease Research and Treatment), Liver Unit, Department of Medical and Surgical Sciences, University of Foggia, Viale Pinto 1, 71122 Foggia, Italy; francesco.cavallone@unifg.it (F.C.); dott.romano@gmail.com (A.D.R.); francesco.bellanti@unifg.it (F.B.); gaetano.serviddio@unifg.it (G.S.)

**Keywords:** liver fibrosis, stiffness, ElastQ, 2D-SWE, chronic hepatitis C, elastography

## Abstract

In recent years, several non-invasive methods have been developed for staging liver fibrosis in patients with chronic hepatitis C. A 2D-Shear wave elastography (SWE) technique has been recently introduced on the EPIQ 7 US system (ElastQ), but its accuracy has not been validated in patients with chronic hepatitis C virus (HCV) infection. We enrolled 178 HCV patients to assess their liver fibrosis stage with ElastQ software using transient elastography as a reference standard. The best cut-off values to diagnose ≥ F2, ≥ F3, and F4 were 8.15, 10.31, and 12.65 KPa, respectively. Liver stiffness values had a positive correlation with transient elastography (*r* = 0.57; *p* < 0.001). The area under the receiver operating characteristics (AUROC) was 0.899 for ≥ F2 (moderate fibrosis), 0.900 for ≥ F3 (severe fibrosis), and 0.899 for cirrhosis. 2D-SWE has excellent accuracy in assessing liver fibrosis in patients with chronic hepatitis C and an excellent correlation with transient elastography.

## 1. Introduction

Chronic hepatitis C is a major cause of end-stage liver disease and health problems globally [1]. Worldwide, an estimated 71 million people have chronic hepatitis C virus (HCV) infections (1.0% of the global population), whereas a higher prevalence has been reported in Europe (more than 14 million people and a prevalence of 1.5%) [2].

The most important clinical feature of an HCV infection is the slowly progressive disease, characterized by persistent hepatic inflammation, fibrosis progression, and finally cirrhosis in up to 50% of patients over 22 years [3,4,5]. In this clinical setting, the assessment of liver fibrosis is a pivotal step for the management of chronic HCV patients since it provides information guiding clinical choices and therapeutic decisions [6,7].

Liver biopsy has always been the gold standard for staging liver fibrosis [8]; however, samples might not be representative of the heterogeneous distribution of hepatic fibrosis in the whole liver [8,9], and limited accuracy due to intraobserver and interobserver variability has also been widely reported [10]. Moreover, liver biopsy is an invasive procedure associated with discomfort and rare but potentially life-threatening complications [11,12]. Therefore, several methods have been studied for the non-invasive assessment of liver fibrosis, including serum biomarkers and quantification of liver stiffness (LS).

Transient elastography (TE) is the standard for the measurement of liver stiffness because it is a well validated and easy to use method, however several other liver elasticity-based imaging techniques have been developing, including ultrasound-based techniques. They are available on a conventional ultrasound machine and allow elastography to be performed during the US examination [13].

Unlike TE, ultrasound-based techniques provide a B-mode image allowing the operator to choose the area on which to perform the stiffness measurements.

By using the trackball of the US system, the user avoids large blood vessels and artifacts, lowering the failure rate and improving the non-invasive approach for the assessment of liver fibrosis.

Ultrasound elastography includes several techniques; however, in the field of liver elastography, the most used methods are the point shear wave elastography (pSWE) and two-dimensional shear wave elastography (2D-SWE) [8,13]. 

Currently, only a few studies have evaluated 2D-SWE for the staging of liver fibrosis in patients with chronic hepatitis C, and definitive cut-off values are not available [8]. A 2D-SWE technique has been recently introduced on the EPIQ 7 (ElastQ) US system (Philips Medical System, Best, the Netherlands), but data on its diagnostic accuracy in predicting fibrosis stages are insufficient. Therefore, we studied a cohort of HCV patients to investigate the accuracy of liver stiffness measurement by 2D-SWE in the staging of liver fibrosis and its correlation with TE.

## 2. Materials and Methods 

### 2.1. Patients and Study Design

We enrolled 178 HCV patients who were referred to the Liver Unit of C.U.R.E. (University Centre for Liver Disease Research and Treatment) at the University of Foggia between January 2017 and February 2020. All patients signed a written informed consent document to participate in the study. The study was conducted according to the principles reported in the Declaration of Helsinki and approved by the authors’ institutional review board.

The inclusion criteria were:

- age ≥18 years
- history of chronic HCV infection treated with Direct-acting antivirals (DAAs)
- undetectable HCV RNA for at least 6 months after DAA treatment completion
- annual TE starting from at least 6 months after DAA treatment completion
- at least 3 evaluations of liver stiffness by TE
- liver stiffness by TE showing stable fibrosis stage over the last 36 months

Exclusion criteria were:

- active HCV infection
- alanine transaminases (ALT) and/or aspartate transaminases (AST) and or gamma-glutamyltransferase (GGT) > 100 U/L over the last 36 months
- presence of ascites, jaundice or hepatocellular carcinoma
- hepatitis B virus (HBV) or human immunodeficiency virus (HIV) coinfection
- liver steatosis at the basic US examination
- daily alcohol intake ≥ 30 g in men and ≥ 20 g in women
- congestive heart failure
- BMI > 30 Kg/m^2^
- concomitant autoimmune liver disease or storage diseases
- diagnosis of drug-induced liver injury (DILI) in the last 36 months.

All patients underwent on the same day:

- B-mode abdominal US
- liver stiffness evaluation by TE (Fibroscan)
- liver stiffness evaluation by 2D-SWE (ElastQ)

According to the EASL guidelines for the assessment of liver fibrosis in patients with chronic hepatitis C [8], TE is the standard for the measurement of liver stiffness, and a combination of TE + serum biomarkers can be used for the identification of patients with liver cirrhosis (F4) or severe fibrosis (F3) without the need for a liver biopsy [8].

Stiffness values reported by Arena et al. were used for staging liver fibrosis during TE [14]. A cut-off value of 14.8 KPa and 10.8 KPa indicated liver cirrhosis and F3 stage of liver fibrosis, respectively. Data from patients without fibrosis (F0) or with mild fibrosis (F1) were combined and considered as one group because they were not clinically different. Patients with liver stiffness < 7.8 KPa were included in this group [14]. Patients with F2 stage of fibrosis had liver stiffness between 7.8 and 10.8 KPa. 

### 2.2. Abdominal Ultrasonography, Transient Elastography, and Two-Dimensional Shear Wave Elastography

Abdominal US, TE, and 2D-SWE were performed after an 8–10 h fast. Abdominal US and 2D-SWE examinations were performed using the EPIQ7 US system with ElastQ software (Philips, Medical System, Best, the Netherlands) and a C1-6 convex probe.

2D-SWE is currently the newest SWE method that is based on the combination of acoustic radiation force by focused ultrasonic beams, and a very high frame ultrasound imaging, recording in real time the propagation of shear waves [8,15]. The size and location of the region of interest (ROI) can be chosen by the operator. A real-time 2D color map, called an elastogram, is superimposed on the B-mode image and, at the same time, a confidence map is displayed to improve the accuracy and quality of the stiffness measurement [16]. The examinations were performed on the right lobe by an intercostal approach with the ROI positioned 3–7 cm below the skin surface, avoiding vessels, rib shadows, and liver/kidney interface. The patient has his/her right arm in maximum abduction in a neutral position avoiding a deep breath, Valsalva maneuver, or deep expiration, which could impact the stiffness measurements, changing the hepatic venous pressures [17,18,19]. Each measurement was performed using an ROI of 1 cm in diameter, and only examinations with ten valid measurements were considered. For each measurement, the median value and interquartile range (IQR) were expressed as kilopascal (KPa). 

Transient elastography was performed by Fibroscan (Echosense, Paris) on a patient lying supine with their arm elevated. The tip of the probe is contacted to the intercostal space at the level of the right lobe. The probe includes an ultrasound transducer and a mechanical device. The mechanical device provides a controlled vibrating external shot on the body surface to generate shear waves. TE measures LS in a volume that is a cylinder 1 cm wide between 25 mm and 65 mm (M probe) or 35 mm and 75 mm (XL probe) below the skin surface. Criteria for a valid examination were:

- at least 10 valid measurements
- a success rate (the ratio of valid measurements to the total number of valid and invalid measurements) above 60%
- IQR less than 30% of the median value.

Measurements were expressed as KPa [8,16,20].

### 2.3. Clinical and Laboratory Assessment

All patients were also evaluated with clinical and laboratory examinations. The clinical approach included a physical examination and anthropometric tests. Laboratory assessment included platelets, alanine transaminases (ALT), aspartate transaminases (AST), gamma-glutamyltransferase (GGT). 

### 2.4. Statistical Analysis

Statistical analysis was performed using SPSS, Version 24 (IBM, Armonk, NY, USA) and GraphPad Prism version 7 (GraphPad Software, La Jolla California USA). Categorial data were reported as absolute numbers (percentages) and continuous variables as mean ± standard deviation (SD). Spearman’s correlation coefficient was used to assess the agreement between the LS acquired by TE and 2D-SWE. Differences among median values of stiffness in different liver fibrosis stages were performed by a Kruskal––Wallis test and Dunn’s multiple comparison test. Receiver operating characteristics (ROC) curves for 2D-SWE were performed. Cut-off values were determined for 2D-SWE after the estimation of the Youden index. Sensitivity, specificity, positive predictive value (PPV), negative predictive value (NPV), positive likelihood ratio (LR+) and negative likelihood ratio (LR−) were also calculated to study the accuracy of 2D-SWE in predicting liver fibrosis. The reference standard for the fibrosis staging was TE (Fibroscan). Two-tailed p values < 0.05 were considered statistically significant. 

## 3. Results

### 3.1. Baseline Characteristic of Patients

We enrolled 185 HCV patients who underwent a US examination and a 2D-SWE study on the same day. Failure to obtain reliable 2D-SWE measurements was observed in four patients (feasibility 97.8%), whereas unreliable TE measurements were recorded in three patients (feasibility 98.4%), therefore 178 patients were included in the final analysis. TE was performed using an M probe in all patients.

The baseline characteristics of enrolled patients are reported in Table 1. Most patients were male, and about thirty-three percent of them had liver cirrhosis. 

### 3.2. Liver Stiffness in Patients with Chronic Hepatitis C

The median of LS significantly increased by stage (Table 2).

**Figure 1:**
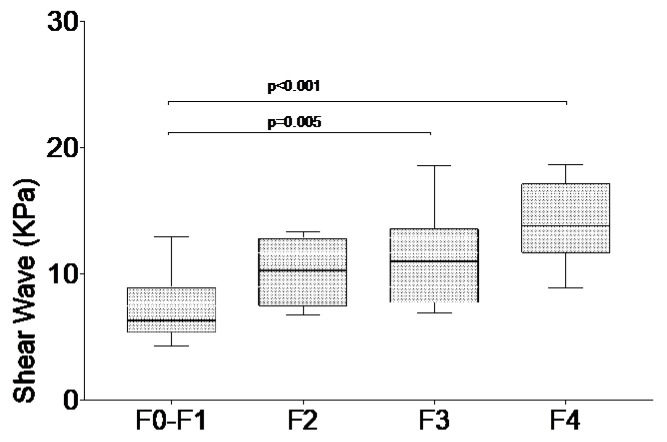
Shear wave elastography values in chronic HCV patients according to fibrosis stage by transient elastography. Values are shown as median and interquartile ranges.

Particularly statistically different values were recorded in those without fibrosis to mild fibrosis (F0-F1) versus the severe fibrosis (F3) group and in F0-F1 versus the liver cirrhosis (F4) group (Figure 1). No differences were observed between intermediate fibrosis stages (F2 vs. F3).

Subgroup analysis by sex and age is reported in Table 2. Older patients showed higher median values of liver stiffness compared with the overall population in F0-F1 and F4 groups, whereas subgroup analysis by sex did not show significant differences in comparison with the overall population. 

Liver stiffness measured by 2D-SWE had a strong and statistically significant positive correlation with liver stiffness measurements obtained with TE (*r* = 0.57; 95% CI 0.35–0.69; *p* < 0.001).

### 3.3. Accuracy of 2D-SWE in Differentiating Different Degrees of Fibrosis

Table 3 summarizes the cut-off values and performance in diagnosing different fibrosis stages of 2D-SWE. The best cut-off values to diagnose ≥ F2, ≥ F3, and F4 stages were 8.15, 10.31, 12.65 KPa, respectively.

Data reported in Table 3 suggest that cut-off values for F3 and F4 stages have a high negative predictive value (NPV) (90.2% and 90.9% respectively) and a moderate increase in the positive likelihood ratio (LR+) value (+5.280 and +6.373 respectively). The best LR+ value (+16.7) for the diagnosis of cirrhosis was recorded when a threshold value of 13 KPa was used. This cut-off value was also associated with an excellent specificity (97.3%), but, on the other hand, sensitivity was very low (45.1%). The cut-off value of 8.15 KPa was associated with the lowest negative likelihood ratio (LR−) value (0.176), low NPV (70.2%) and specificity (73%), and high sensitivity (87.1%). 

Subgroup analysis by age (Table 4) showed a high specificity for all cut-off values and high NPV when 13 KPa is used as a reference for the exclusion of liver cirrhosis.

## 4. Discussion

The assessment of liver fibrosis is a pivotal step in the management of patients with chronic liver disease [21]. Due to the high prevalence of global infection and the natural history of HCV infection, a significant number of HCV patients require an evaluation of liver fibrosis over time, but a liver biopsy cannot be routinely used for the clinical follow-up because of its invasiveness and potentially life-threatening complications [11]. Currently, TE is considered the new gold standard for the assessment of liver fibrosis in HCV patients because it has shown excellent accuracy in staging liver fibrosis. However, this technique has several limitations such as the lack of anatomical image and low applicability in obese individuals or in patients with ascites. Therefore, an improvement of techniques for the non-invasive assessment of liver fibrosis has been a new challenge for the management of patients with liver disease.

2D-SWE has recently been introduced in clinical practice as a very simple, non-invasive, and easy to repeat technique to evaluate liver fibrosis over time.

In the last few years, several companies such as GE, Philips, and Canon, developed 2D-SWE applications for US systems. All provide both stiffness color maps and the opportunity for multiple stiffness measurements within the ROI. Moreover, new SWE-based techniques (such as real-time 2D-SWE) have recently provided a challenging improvement to make faster imaging representations of stiffness color maps and SWE measurements and finally optimize the timing for an ultrasound examination. 

Briefly, there is currently a great interest in SWE applications implemented on US systems aimed at providing useful and simple support for clinicians involved in the management of patients with chronic liver disease.

However, today little data on the clinical applicability of 2D-SWE are available, as ultrasound imaging systems use different techniques, and cut-off levels are not universally applicable.

Data on accuracy of ElastQ (Philips Medical System, Best, the Netherlands) and its correlation with TE in HCV patients are lacking because most data available in HCV patients have been obtained by Fibroscan [22] or different 2D-SWE techniques [13,23,24,25]. For instance, some authors proposed 10 KPa and 15.6 KPa as optimal cut-off values for the identification of significant fibrosis (≥F3) and cirrhosis (F4), respectively, in HCV patients (*N* = 121) [26]. In another large HCV population study, the authors defined 13 KPa as the best cut-off for cirrhosis [7]. However, all of them used a different technique as compared to our study.

We studied a large cohort of HCV patients and estimated the best cut-off values for the exclusion of liver cirrhosis (KPa 12.65; AUROC = 0.899; NPV: 90.9%) and diagnosis of F2 fibrosis (KPa 8.15; AUROC = 0.899; sensitivity 87%; PPV 87.3%). As previously reported by several authors for TE and other 2D-SWE techniques [7,27,28], ElastQ is a very reliable tool to exclude cirrhosis. 

Our cut-off levels for predicting liver fibrosis are very similar to the fibrosis classifications proposed by Bavu et al. [29], showing that different 2D-SWE techniques but the same cut-off levels might be used in HCV patients.

The analysis of liver stiffness in the older population (≥ 65 years) showed higher medians for all fibrosis stages than those reported in the overall population with 13 KPa (AUROC 0.900; specificity 93.3%; NPV 94.3%) as the threshold value for the exclusion of liver cirrhosis. In patients aged ≥ 65 years, the best cut–off value for distinguishing patients with mild or absent fibrosis from those with significant fibrosis (F2 stage or more) was 10.4 KPa, a threshold value that had high specificity (95%) and PPV (92.3%; LR+ 14.2) and finally high accuracy for the diagnosis of significant fibrosis. 

On the other hand, no differences were found between liver stiffness in males and females by fibrosis stage, as previously reported [13,30].

As for liver biopsy, 2D-SWE by ElastQ revealed a low performance for differentiating F2 from F3 stages [31]. We showed that 2D-SWE had a good accuracy in classifying liver fibrosis into three stages (absent/mild fibrosis, intermediate fibrosis, and cirrhosis) without a good performance in distinguishing between F2 and F3 stages. It is noteworthy that we included in our analysis patients without potential confounding factors such as liver steatosis, obesity, concomitant liver disease, and food intake with significant improvement of accuracy of measurements. 

Our data also showed a moderate correlation between 2D-SWE and TE (*r* = 0.57; 95% CI 0.35–0.69; *p* < 0.001). Probably a moderate correlation coefficient could be attributed to the narrow range of stiffness values for low-grade fibrosis versus a wide range for significant fibrosis. Despite that, our results suggest that the assessment of liver stiffness with 2D-SWE may be performed during a routine US examination, simplifying the follow-up of patients with a history of chronic HCV infection. Limitations of our analysis are the small number of patients in each subgroup by fibrosis stage, the single-center design of the study, and the unavailability of a liver biopsy. Therefore, further studies are needed to confirm our results and find definitive and universally applicable cut-off values. 

So far, our data have shown that 2D-SWE performed with ElastQ software has excellent accuracy in the staging of liver fibrosis in HCV patients, and a good correlation with TE. Based on our results, 2D-SWE with ElastQ has proven to be useful for the management of HCV patients not only when TE is not applicable. 

Providing a B-mode imaging and allowing the users to choose the best ROI, 2D-SWE can successfully overcome the limitations of TE and improve the non-invasive approach for the assessment of liver fibrosis. 

## Figures and Tables

**Table 1:** Baseline characteristics of the study population.

All Patients *N* = 178	F0-F1 Group *N* = 44	F2 Group *N* = 32	F3 Group *N* = 43	F4 Group *N* = 59
Age (years)	64.2 ± 13	63.7 ± 13	64.1 ± 12.7	64.5 ± 13
Gender (M/F)	30/14	19/13	22/21	36/23
BMI kg/m^2^	24.1 ± 4.3	23.1 ± 3.7	24.5 ± 4.1	23.8 ± 2.9
Aspartate transaminases (AST) (U/L)	32.4 ± 16.8	38.2 ± 23.3	29.9 ± 17.3	26.2 ± 24.1
Alanine transaminases (ALT) (U/L)	19.2 ± 11.3	31.3 ± 26.8	39.4 ± 13.8	38.6 ± 19.8
Gamma-glutamyltransferase (GGT) (U/L)	22.3 ± 19.7	31.1 ± 18.6	36.8 ± 21.3	37.2 ± 9.9

**Table 2:** Liver Stiffness obtained with ElastQ software by subgroups.

Liver Stiffness (ElastQ)	F0–F1 Group	F2 Group	F3 Group	F4 Group
**Overall Population (*N* = 178)**				
Median (KPa)	6.31	10.28	11.03	13.83
25% Percentile	5.36	7.47	7.76	11.72
75% Percentile	8.94	12.81	13.6	17.15
**Female (*N* = 71)**				
Median (KPa)	6.17	10.23	11.52	14.40
25% Percentile	5.40	7.06	7.96	10.91
75% Percentile	9.83	12.39	12.2	15.90
**Male (*N* = 107)**				
Median (KPa)	6.39	9.1	11.47	13.58
25% Percentile	5.35	6.8	9.55	12
75% Percentile	7.51	10.4	18.56	18.22
**≥** **65 years (*N* = 97)**				
Median (KPa)	7.22	10.06	10.55	15.07
25% Percentile	5.38	6.8	8.01	12.57
75% Percentile	9.2	13.39	12.47	17.52

**Table 3:** Diagnostic performance of 2D-SWE (ElastQ) in the overall population.

Fibrosis Stage	Cut-off Value (KPa)	AUROC (95% CI)	Sensitivity % (95% CI)	Specificity % (95% CI)	PPV % (95% CI)	NPV % (95% CI)	LR+	LR−
**F = 4**	12.65	0.899 (0.824–0.973)	73.3 (44.9–92.2)	88.5 (76.6–95.7)	69.5 (54.2–89.7)	90.9 (82.3–99.7)	6.373	0.301
**F ≥ 3**	10.31	0.900 (0.834–0.967)	77.1 (59.9–89.6)	85.4 (70.8–94.4)	77.7 (61.6–97.3)	90.2 (80.7–99.9)	5.280	0.268
**F ≥ 2**	8.15	0.899 (0.829–0.969)	87.1 (70.2–96.4)	73 (55.9–86.2)	87.3 (70.4–99.9)	70.2 (58.3–96.9)	3.223	0.176

**Table 4:** Diagnostic performance of 2D-SWE (ElastQ) in patients aged ≥65 years.

Fibrosis Stage	Cut–off Value (KPa)	AUROC (95% CI)	Sensitivity % (95% CI)	Specificity % (95% CI)	PPV % (95% CI)	NPV % (95% CI)	LR+	LR−
**F = 4**	13.00	0.900 (0.781–1.000)	71.4 (39–96.3)	93.3 (77.9–99.2)	73.6 (48–99.8)	94.3 (84.4–99.9)	10.7	0.116
**F ≥ 3**	11.29	0.847 (0.722–0.973)	64.3 (35.2–87.2)	91.3 (72–98.9)	81.8 (61.6–100)	80.8 (64.7–96.9)	7.39	0.183
**F ≥ 2**	10.47	0.862 (0.744–0.979)	70.6 (44.1–89.7)	95 (75.1–99.9)	92.3 (77.8–100)	79.2 (63–95.4)	14.2	0.071

## References

[1] World Health Organization (WHO) Hepatitis C. https://www.who.int/news-room/fact-sheets/detail/hepatitis-c.

[2] Han R., Zhou J., Francois C., Toumi M. (2019). Prevalence of hepatitis C infection among the general population and high-risk groups in the EU/EEA: A systematic review update. BMC Infect. Dis..

[3] Westbrook R.H., Dusheiko G. (2014). Natural history of hepatitis C. J. Hepatol..

[4] Tong M.J., el-Farra N.S., Reikes A.R., Co R.L. (1995). Clinical outcomes after transfusion-associated hepatitis C. N. Engl. J. Med..

[5] Wiese M., Berr F., Lafrenz M., Porst H., Oesen U. (2000). Low frequency of cirrhosis in a hepatitis C (genotype 1b) single-source outbreak in germany: A 20-year multicenter study. Hepatology.

[6] Lefton H.B., Rosa A., Cohen M. (2009). Diagnosis and epidemiology of cirrhosis. Med. Clin. N. Am..

[7] Herrmann E., de Ledinghen V., Cassinotto C., Chu W.C., Leung V.Y., Ferraioli G., Filice C., Castera L., Vilgrain V., Ronot M. (2018). Assessment of biopsy-proven liver fibrosis by two-dimensional shear wave elastography: An individual patient data-based meta-analysis. Hepatology.

[8] European Association for the Study of the Liver, Asociacion Latinoamericana para el Estudio del Higado (2015). EASL-ALEH Clinical Practice Guidelines: Non-invasive tests for evaluation of liver disease severity and prognosis. J. Hepatol..

[9] Regev A., Berho M., Jeffers L.J., Milikowski C., Molina E.G., Pyrsopoulos N.T., Feng Z.Z., Reddy K.R., Schiff E.R. (2002). Sampling error and intraobserver variation in liver biopsy in patients with chronic HCV infection. Am. J. Gastroenterol..

[10] Bedossa P., Dargere D., Paradis V. (2003). Sampling variability of liver fibrosis in chronic hepatitis C. Hepatology.

[11] Castera L., Negre I., Samii K., Buffet C. (1999). Pain experienced during percutaneous liver biopsy. Hepatology.

[12] Bravo A.A., Sheth S.G., Chopra S. (2001). Liver biopsy. N. Engl. J. Med..

[13] Serra C., Grasso V., Conti F., Felicani C., Mazzotta E., Lenzi M., Verucchi G., D’Errico A., Andreone P. (2018). A New Two-Dimensional Shear Wave Elastography for Noninvasive Assessment of Liver Fibrosis in Healthy Subjects and in Patients with Chronic Liver Disease. Ultraschall Med..

[14] Arena U., Vizzutti F., Abraldes J.G., Corti G., Stasi C., Moscarella S., Milani S., Lorefice E., Petrarca A., Romanelli R.G. (2008). Reliability of transient elastography for the diagnosis of advanced fibrosis in chronic hepatitis C. Gut.

[15] Muller M., Gennisson J.L., Deffieux T., Tanter M., Fink M. (2009). Quantitative viscoelasticity mapping of human liver using supersonic shear imaging: Preliminary in vivo feasibility study. Ultrasound Med. Biol.

[16] Sigrist R.M.S., Liau J., Kaffas A.E., Chammas M.C., Willmann J.K. (2017). Ultrasound Elastography: Review of Techniques and Clinical Applications. Theranostics.

[17] Barr R.G., Ferraioli G., Palmeri M.L., Goodman Z.D., Garcia-Tsao G., Rubin J., Garra B., Myers R.P., Wilson S.R., Rubens D. (2015). Elastography Assessment of Liver Fibrosis: Society of Radiologists in Ultrasound Consensus Conference Statement. Radiology.

[18] Yun M.H., Seo Y.S., Kang H.S., Lee K.G., Kim J.H., An H., Yim H.J., Keum B., Jeen Y.T., Lee H.S. (2011). The effect of the respiratory cycle on liver stiffness values as measured by transient elastography. J. Viral Hepat..

[19] Horster S., Mandel P., Zachoval R., Clevert D.A. (2010). Comparing acoustic radiation force impulse imaging to transient elastography to assess liver stiffness in healthy volunteers with and without valsalva manoeuvre. Clin. Hemorheol. Microcirc..

[20] Castera L., Forns X., Alberti A. (2008). Non-invasive evaluation of liver fibrosis using transient elastography. J. Hepatol..

[21] Ferraioli G. (2019). Review of Liver Elastography Guidelines. J. Ultrasound Med..

[22] Ferraioli G., Wong V.W., Castera L., Berzigotti A., Sporea I., Dietrich C.F., Choi B.I., Wilson S.R., Kudo M., Barr R.G. (2018). Liver Ultrasound Elastography: An Update to the World Federation for Ultrasound in Medicine and Biology Guidelines and Recommendations. Ultrasound Med. Biol..

[23] Abe T., Kuroda H., Fujiwara Y., Yoshida Y., Miyasaka A., Kamiyama N., Takikawa Y. (2018). Accuracy of 2D shear wave elastography in the diagnosis of liver fibrosis in patients with chronic hepatitis C. J. Clin. Ultrasound.

[24] Matos J., Paparo F., Bacigalupo L., Cenderello G., Mussetto I., De Cesari M., Bernardi S.P., Cevasco L., Forni G.L., Cassola G. (2019). Noninvasive liver fibrosis assessment in chronic viral hepatitis C: Agreement among 1D transient elastography, 2D shear wave elastography, and magnetic resonance elastography. Abdom. Radiol. (NY).

[25] Ferraioli G., Maiocchi L., Lissandrin R., Tinelli C., De Silvestri A., Filice C. (2016). Liver Fibrosis Study Group, Accuracy of the ElastPQ Technique for the Assessment of Liver Fibrosis in Patients with Chronic Hepatitis C: A “Real Life” Single Center Study. J. Gastrointestin. Liver Dis..

[26] Ferraioli G., Tinelli C., Dal Bello B., Zicchetti M., Filice G., Filice C., Liver Fibrosis Study G. (2012). Accuracy of real-time shear wave elastography for assessing liver fibrosis in chronic hepatitis C: A pilot study. Hepatology.

[27] Friedrich-Rust M., Ong M.F., Martens S., Sarrazin C., Bojunga J., Zeuzem S., Herrmann E. (2008). Performance of transient elastography for the staging of liver fibrosis: A meta-analysis. Gastroenterology.

[28] Tsochatzis E.A., Gurusamy K.S., Ntaoula S., Cholongitas E., Davidson B.R., Burroughs A.K. (2011). Elastography for the diagnosis of severity of fibrosis in chronic liver disease: A meta-analysis of diagnostic accuracy. J. Hepatol..

[29] Bavu E., Gennisson J.L., Couade M., Bercoff J., Mallet V., Fink M., Badel A., Vallet-Pichard A., Nalpas B., Tanter M. (2011). Noninvasive in vivo liver fibrosis evaluation using supersonic shear imaging: A clinical study on 113 hepatitis C virus patients. Ultrasound Med. Biol..

[30] Popescu A., Sporea I., Sirli R., Bota S., Focsa M., Danila M., Nicolita D., Martie A., Sendroiu M., Juchis A. (2011). The mean values of liver stiffness assessed by Acoustic Radiation Force Impulse elastography in normal subjects. Med. Ultrason..

[31] Poynard T., Lenaour G., Vaillant J.C., Capron F., Munteanu M., Eyraud D., Ngo Y., M’Kada H., Ratziu V., Hannoun L. (2012). Liver biopsy analysis has a low level of performance for diagnosis of intermediate stages of fibrosis. Clin. Gastroenterol. Hepatol..

